# Supplementary material for: Natural Extracts Mitigate the Deleterious Effects of Prolonged Intense Physical Exercise on the Cardiovascular and Muscular Systems
Source: Antioxidants (Basel). 2023 Jul 22;12(7):1474. doi: 10.3390/antiox12071474 (PMC10376415; doi:10.3390/antiox12071474)
Supplement: Supplementary file 1 [file antioxidants-12-01474-s001.zip › antioxidants-2480809-supplementary.pdf]

Supplemental Figure S1

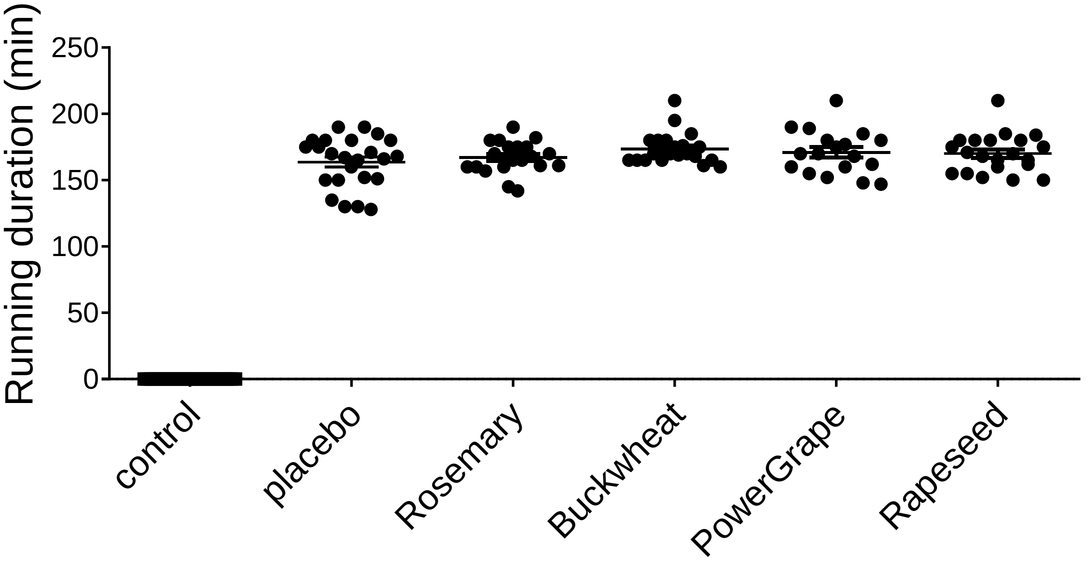

Supplemental Figure S2

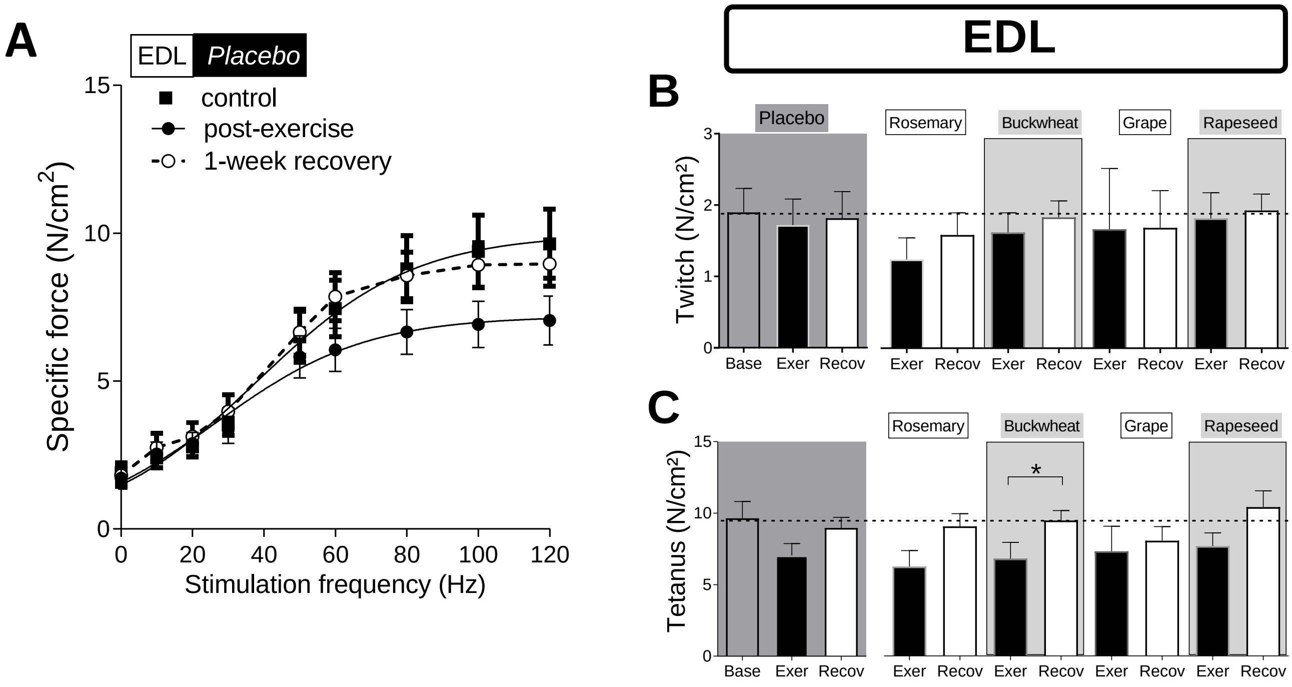

Figure S2. Effects of the 4 different natural extracts on the isometric strength of EDL after exhausting exercise and one week of recovery. (A) Force–frequency relationships of EDL muscles measured *in vitro* from Sedentary (black square), 30 min post-exercise (black circle) and after one week of recovery (open circle, dash line) placebo groups. Results are expressed as mean ± SEM, (*n*=10-13 animals/group). *P < 0.05 Placebo post-exercise *vs* Basal. **(B-C)** reveal the different contractile characteristics measured *in vitro* of the EDL muscles in groups of animals fed with the 4 different natural extracts (Rosemary, Buckwheat, Powergrape and Rapeseed) either just post-exercise or one- week later. **B** represent the force induced by a single twitch, and **C** represent the tetanic contraction or peak specific force. Results are expressed as mean ±SEM, (*n*=7-13 in each group). $ P < 0.05 *vs* basal, *P < 0.05 Recovery *vs* Post-exercise.

Supplemental Figure S3

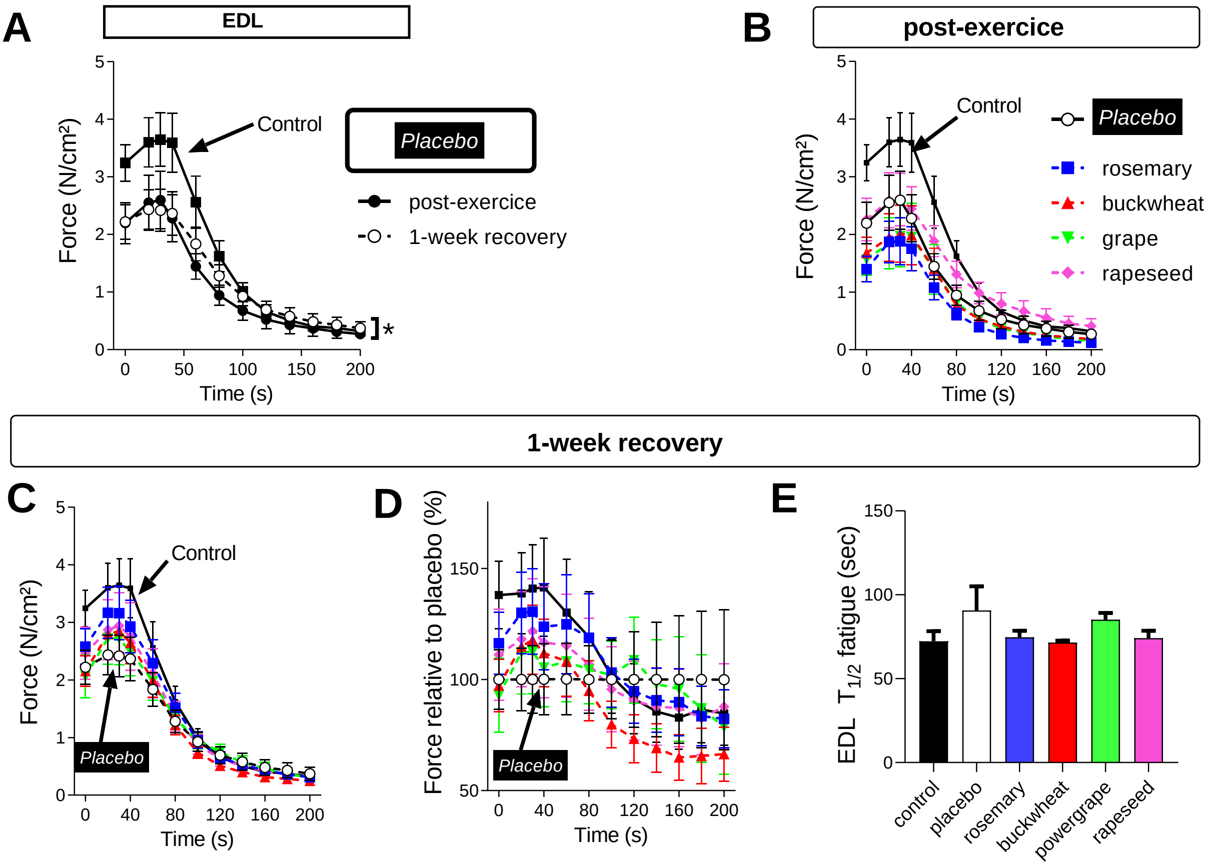

Figure S3. Effects of the 4 different natural extracts on the EDL fatigability after exercise and recovery. (A) Test of fatigue *in vitro* on soleus muscles in sedentary rats (control, black square), placebo post-exercise (black circle) and placebo one-week recovery (open circle) groups. **B)** Effects of Rosemary (blue square), Buckwheat (red triangle), Powergrape (green triangle) and Rapeseed (pink diamond) on *in vitro* post-exercise muscle fatigue test in soleus compared with placebo (open circle) and controls (black square). **C-E)** Effects of the natural extracts on the *in vitro* muscle fatigue test one week after exercise compared with placebo (open circle) and controls (black square). **D)** Muscle fatigue was also expressed relative to the level of force of the placebo group at each time point. **E)** Time to 1/2 loss of maximum strength during fatigue test in the different groups after one week of recovery. (*n*=7-13 animals per group). *P < 0.05 placebo *vs* Control, Rosemary, Buckwheat, Powergrape and Rapeseed groups.

Table S1: ECG parameters before, 30-min after and 7 days after intense exercise in presence or absence of antioxidant naturel extracts.

		PR duration (ms)			QRS duration (ms)		
		Base	Post-Ex	Recov	Base	Post-Ex	Recov
placebo	(n=8)	43.7±2.9	42.0±2.6	44.3±3.4	22.1±2.2	22.7±1.8	23.0±2.7
rosemary	(n=8)	45.0±2.9	44.1±4.4	45.4±4.0	24.9±1.7	22.7±2.4	24.3±3.0
bucklewheat	(n=9)	43.0±1.6	41.7±2.2	42.2±2.2	22.0±2.1	20.2±2.0	22.8±2.0
powergrape	(n=7)	44.2±3.0	43.1±3.5	44.0±3.0	21.8±2.6	19.9±4.1	23.5±2.5
rapeseed	(n=8)	45.8±1.5	44.0±3.4	45.2±2.5	22.6±2.1	21.6±3.7	23.4±1.6
